# Inhibition of 2-arachidonoylglycerol degradation enhances glial immunity by single-cell transcriptomic analysis

**DOI:** 10.1186/s12974-023-02701-4

**Published:** 2023-01-30

**Authors:** Dexiao Zhu, Jian Zhang, Jack Hashem, Fei Gao, Chu Chen

**Affiliations:** 1 Department of Cellular and Integrative Physiology, Joe R. and Teresa Lozano Long School of Medicine, University of Texas Health Science Center at San Antonio, 7703 Floyd Curl Drive, San Antonio, TX 78229 USA; 2 Center for Biomedical Neuroscience, Joe R. and Teresa Lozano Long School of Medicine, University of Texas Health Science Center at San Antonio, San Antonio, TX 78229 USA

**Keywords:** Endocannabinoid, Immunity, Neuroinflammation, Monoacylglycerol lipase, Transcriptome, Single-cell RNA sequencing, Neurodegenerative disease, Vigilance

## Abstract

**Background:**

2-Arachidonoylglycerol (2-AG) is the most abundant endogenous cannabinoid. Inhibition of 2-AG metabolism by inactivation of monoacylglycerol lipase (MAGL), the primary enzyme that degrades 2-AG in the brain, produces anti-inflammatory and neuroprotective effects in neurodegenerative diseases. However, the molecular mechanisms underlying these beneficial effects are largely unclear.

**Methods:**

Hippocampal and cortical cells were isolated from cell type-specific MAGL knockout (KO) mice. Single-cell RNA sequencing was performed by 10 × Genomics platform. Cell Ranger, Seurat (v3.2) and CellChat (1.1.3) packages were used to carry out data analysis.

**Results:**

Using single-cell RNA sequencing analysis, we show here that cell type-specific MAGL KO mice display distinct gene expression profiles in the brain. Inactivation of MAGL results in robust changes in expression of immune- and inflammation-related genes in microglia and astrocytes. Remarkably, upregulated expression of chemokines in microglia is more pronounced in mice lacking MAGL in astrocytes. In addition, expression of genes that regulate other cellular functions and Wnt signaling in astrocytes is altered in MAGL KO mice.

**Conclusions:**

Our results provide transcriptomic evidence that cell type-specific inactivation of MAGL induces differential expression of immune-related genes and other fundamental cellular pathways in microglia and astrocytes. Upregulation of the immune/inflammatory genes suggests that tonic levels of immune/inflammatory vigilance are enhanced in microglia and astrocytes, particularly in microglia, by inhibition of 2-AG metabolism, which likely contribute to anti-inflammatory and neuroprotective effects produced by inactivation of MAGL in neurodegenerative diseases.

**Supplementary Information:**

The online version contains supplementary material available at 10.1186/s12974-023-02701-4.

## Background

Endocannabinoids are endogenous lipid mediators involved in a variety of physiological and pathological processes [1, 2]. The actions of endocannabinoids in these processes are primarily mediated through acting on G-protein coupled cannabinoid receptors (CB_1_ and CB_2_), which are the targets of Δ^9^-tetrahydrocannabinol (Δ^9^-THC), the primary psychoactive ingredient of cannabis or marijuana. 2-Arachidonoylglycerol (2-AG) is the most abundant endocannabinoid and has been recognized as a retrograde messenger modulating synaptic transmission and plasticity at both GABAergic and glutamatergic synapses [3–9]. In particular, 2-AG has been shown to play an important role in maintaining brain homeostasis and protecting neurons from harmful insults in both in vitro and in vivo [10–17]. However, 2-AG is an unstable lipid and is easily broken down by several enzymes, including monoacylglycerol lipase (MAGL), α/β hydrolase domain-containing protein 6 and 12 (ABHD6/12), cyclooxygenase-2 (COX-2), cytochromes, and lipoxygenases [2, 18–20]. To augment 2-AG signaling, an optimal strategy is to inhibit the enzymatic activity that degrades 2-AG. It has been estimated that approximate 85% of 2-AG in the brain is hydrolyzed by MAGL [19, 20], suggesting that MAGL is the primary enzyme degrading 2-AG in the brain. Indeed, 2-AG levels in the brain are robustly elevated by pharmacological or genetic inactivation of MAGL, while 2-AG metabolites arachidonic acid (AA) and prostaglandins (PGs), which are proinflammatory, are greatly reduced [19–24].

Previous studies provided evidence that pharmacological or genetic inactivation of MAGL alleviates neuropathology and improves synaptic and cognitive functions in several animal models of neurodegenerative diseases [21, 23–28]. Thus, it has been proposed that MAGL is a therapeutic target for neurodegenerative diseases, including Alzheimer’s disease, amyotrophic lateral sclerosis, multiple sclerosis, Parkinson’s disease, and traumatic brain injury [23–25, 29–33]. However, our understanding of the molecular mechanisms responsible for neuroprotective effects of MAGL inactivation in neurodegenerative diseases is still limited. Inflammation has been recognized as a common mechanism of disease and neuroinflammation is the root cause of neurodegenerative diseases [34, 35]. It is likely that the neuroprotective effects of MAGL inactivation are mediated through resolving neuroinflammation, which, in turn, mitigates neuropathology and prevents synaptic and cognitive declines in neurodegenerative diseases [36]. To understand the molecular mechanisms of MAGL inactivation in curbing neuroinflammation, we used single-cell transcriptomic analysis of microglia and astrocytes in MAGL conditional knockout (KO) mice, as microglia and astrocytes in the brain are the main players in neuroinflammatory responses and in endocannabinoid signaling-mediated resolution of neuroinflammation [37, 38]. We here show that cell type-specific MAGL knockout (KO) mice display distinct gene expression profiles in the brain. Inactivation of MAGL results in significant changes in immune- and inflammation-related genes in microglia and astrocytes. Importantly, upregulation of chemokines in microglia is more pronounced in astrocytic MAGL KO mice. Our study provides transcriptomic evidence that inactivation of MAGL alters expression profiles of genes that regulate immune, inflammation, oxidative stress, and neuronal functions in microglia and astrocytes. Upregulation of inflammation-related genes in microglia by inactivation of MAGL suggests that inhibition of 2-AG metabolism in astrocytes promotes tonic alertness in both microglia and astrocytes, particularly in microglia, which likely contribute to anti-inflammatory and neuroprotective effects of MAGL inactivation in neurodegenerative diseases.

## Methods

### Animals

*Mgll*^*flox/flox*^ mice were generated by the Texas A&M Institute for Genomic Medicine and bred with different cre mice to produce total and cell type-specific MAGL knockout (KO) mice, as described previously [39]. Briefly, *mgll*^flox/flox^ mice were crossed with Tg(Sox2-cre)1Amc/J (JAX Stock No: 004783) mice to generate total/global MAGL knockouts (tKO). Neuronal and astrocytic MAGL KO mice (nKO and aKO) were generated by crossing *mgll*^flox/flox^ mice with Syn1-cre mice (JAX Stock No: 003966) and GFAP-cre mice (JAX Stock No: 024098), respectively. As demonstrated previously, no changes in level of 2-AG are observed in the brain of microglial-MAGL KO mice [22], and 2-AG in microglia appears to be degraded primarily by ABHD12 [40], suggesting that MAGL in microglial cells may not play an important role in degrading 2-AG. Thus, we did not generate microglial-MAGL KO mice. Both male and female mice at ages of 3 months were used in the presents study. All the animal studies were performed in compliance with the US Department of Health and Human Services Guide for the Care and Use of Laboratory Animals, and approved by the Institutional Animal Care and Use Committee of University of Texas Health San Antonio.

### Single-cell sample preparation

Single-cell suspensions from 3-month-old *mgll*^lox/lox^-non cre (WT), tKO, nKO, and aKO mice were made using an Adult Brain Dissociation Kit (MACS Miltenyi Biotec, Cat# 130-107-677) according to the instructions provided by the manufacturer with some modification [41]. Briefly, mice were anesthetized with ketamine/xylazine (200/10 mg/kg) and the brains were immediately removed and washed in ice-cold D-PBS. The hippocampus and cortex from two animals per group were dissected out and cut as 200 mm slices in Enzyme mix 1. The slices were transferred into T25 flasks containing Enzyme mix 2 and incubated at 37 °C for 25 min. The enzymatically digested cells were transferred into 15-ml tubes with cold D-PBS and centrifuged briefly. The samples at the bottom of the tubes were resuspended with D-PBS and the supernatants were transferred to MACS SmartStrainer (70 m) on 50 ml tubes. After repeated twice, suspensions were centrifuged at 300×*g* for 10 min at 4 °C. The pellets were carefully resuspended in cold D-PBS and final cell suspensions were obtained following removal of dead cells and debris with the cold Debris Removal Solution and removal of blood cells with the cold Red Blood Cell Removal Solution according to the procedures instructed by the manufacture.

### Single-cell RNA sequencing library preparation

Single-cell suspensions were loaded into the 10 × Genomics Chromium microfluidic chips with the intention of capturing 10,000 cells within individual Gel Beads-in-emulsion (GEM). Inside the GEMs the cells were lysed and their RNA was reverse transcribed using a poly(dT) priming. During reverse transcription Cell Barcodes and Unique Molecular Identifiers (UMI) were added to each of the cDNA transcripts. The libraries were prepared for sequencing following the manufacturer’s recommendations for the 10 × Genomics 3′ Gene Expression v3 chemistry as described previously, and sequenced at the North Texas Genome Center located at UT Arlington on an Illumina NovaSeq S4 150 PE flow cell.

### Single-cell RNA-seq data processing

ScRNA data analysis was carried out using the Cell Ranger and Seurat Package (v 3.2) in R (v. 4.0.3), as described previously [39, 42]. For quality control, low-quality cells were excluded from downstream analysis based on the percentage of mitochondrial genes > 30%. Genes detected in fewer than 10 cells were removed. Cells were filtered with following parameters: maximum of nGenes = 6500, minimum of nGenes = 200, maximum of UMIs = 40,000. After filtering, 67,452 cells were retained.

We used the log NormalizeData function to normalize the feature expression measurements from different experiments with a scaling factor of 10,000. The data were scaled using ScaleData function. Most variable features for each object were identified via Find Variable Features function in Seurat. The n features parameter is 3000 and the selection method is ‘vst’. These genes were used for further analysis. To reduce the dimensionality of the dataset, principal component analysis was carried out based on the top 3000 most variable genes using RunPCA function. The top 20 principal components (PCs) were used to cluster cells by Find Cluster function, and the resolution is 1.0. We used a non-linear dimensional reduction method, t-distributed stochastic neighbor embedding (tSNE), to visualize these datasets.

### Classification of cell types

At least three cell type-specific markers, which have been previously reported in the literature, were used to determine cell-type identity. We identified a large population of aqp4-expressed astrocytes. Although *gfap* is a commonly used astrocyte-specific marker, we found expression of *gfap* was restricted to a small number of cells. So *gja1* and *slc1a2* were also used as second and third astrocyte-specific markers in our study. The identity of microglia was marked according to the expression of *aif1*, *itgam*, and *tmem119*.

### Differentially expressed genes analysis

After quality control preprocessing and classification of cellular identities, we performed differential expressed genes analysis using Find Markers function in Seurat (v3.2). The software generated averaged log2 (fold change) of gene expression, the percentage of cells expressing the genes in each group (pct.1 and pct.2), *P* value, and adjusted *P* value using a Wilcoxon method. Min.pct = 0 and logfc. threshold = 0 were used in finding DEGs. Only genes with *P* < 0.05 and log_2_ (fold change) > 0.1 or < − 0.1 were considered as differential expressed genes.

### Heatmap, Venn plot, and GO analysis

Org.Mm.eg.db, clusterProfiler, DAVID 6.8, and ggplot2 packages in R (4.0.3) were used to produce bar-plot of GO terms. All detected genes in specific cell types were used as background. We used Venn Diagram, EnhancedVolcano, and ggplot2 packages in R (4.0.4) to produce Venn plots. Heatmaps were generated to display differential expressed genes (DEGs) in at least one group.

### Cell–cell communication analysis

CellChat package (1.1.3) in R (4.0.4) was used to analyze the communications between astrocytes and microglia according to the guideline [43]. Single-cell RNA-seq data were imported into software after being analyzed by Seurat.

## Results

### Gene expression profiles in MAGL KO mice

**Fig. 1:**
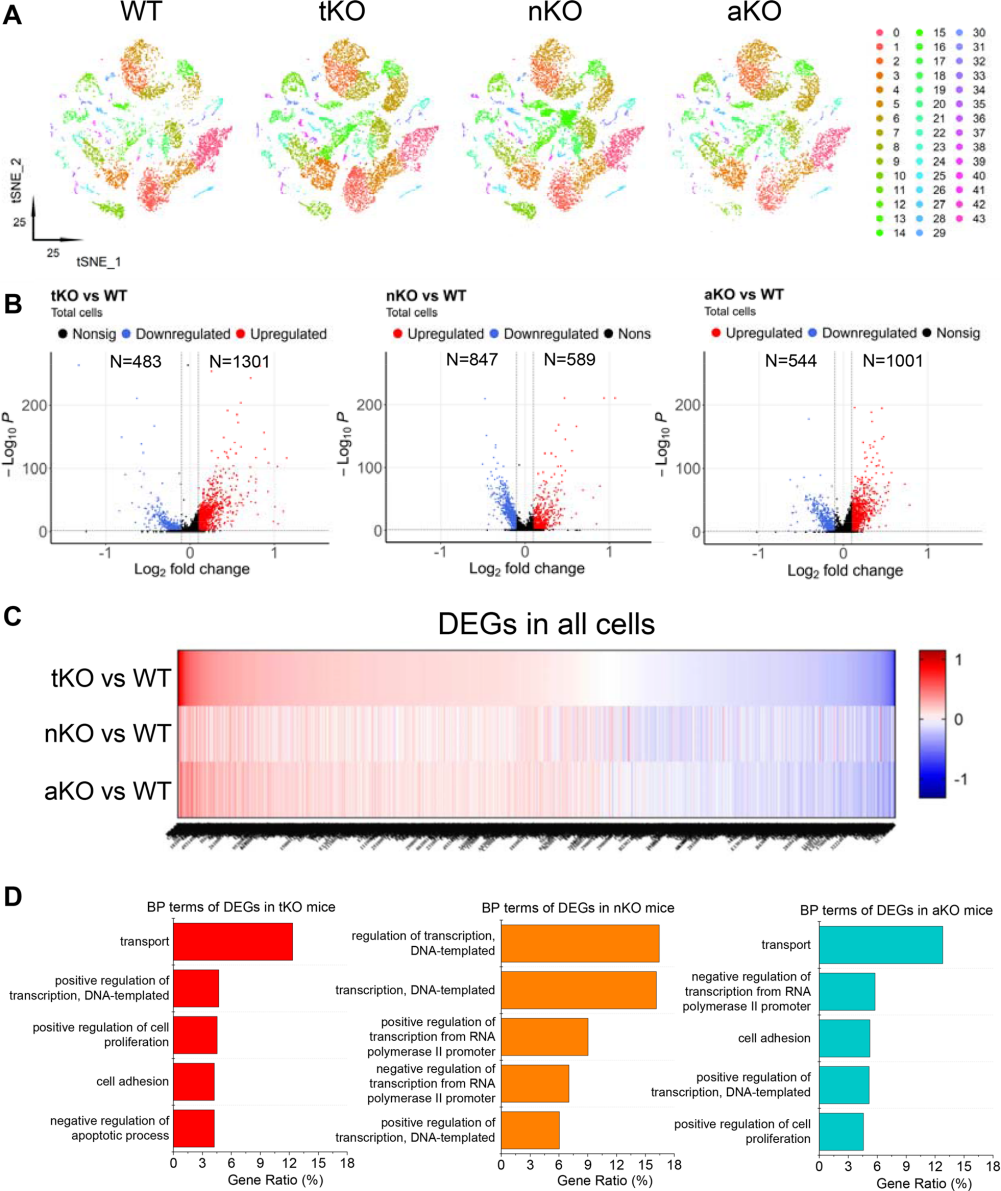
Transcriptome-based cell classification in mouse cortex and hippocampus. **A** T-distributed stochastic neighbor embedding (t-SNE) plots showing the cell clusters in wild type (WT), total MAGL knockout (tKO), neuronal MAGL KO (nKO), and astrocytic MAGL KO (aKO) mice. **B** Volcano plots display up- and down-regulated differentially expresses genes (DEGs) in the brain from different genotypes. **C** Heatmap shows DEGs in the brain of tKO, nKO, and aKO mice. **D** Gene Ontology (GO) analysis of biological process (BP) terms of DEGs in different MAGL KO mice

Previous studies provided evidence that pharmacological or genetic inactivation of MAGL alleviates neuropathology and improves synaptic and cognitive functions in several animal models of neurodegenerative diseases [21, 23–28]. To determine the cellular and molecular mechanisms underlying these beneficial effects of MAGL inactivation, we profile expression of genes in microglia and astrocytes from tKO, nKO and aKO mice to compare with WT mice using 10 × genomics chromium single-cell RNA sequencing analysis. A total of 67,452 cells passed the quality control. To visualize the genes relationship between cells, an unsupervised learning method, t-distributed stochastic neighbor embedding (t-SNE) was used to arrange barcodes in two dimensions. The resulting tSNE plots revealed 43 distinct clusters across all cells (Fig. 1A). Figure 1B shows the numbers of differentially expressed genes (DEGs) in tKO, nKO, and aKO *versus* WT. We identified that there are more upregulated DEGs in tKO and aKO mice, while there are more downregulated DEGs in nKO mice. Heatmap and Venn plots that display the distribution and average Log_2_ (fold change) of DEGs also show the differences between cell type-specific MAGL KO mice (Fig. 1C and Additional file 1: Fig. S1). Figure 1D lists top 5 terms of influenced biological processes (BP) based on comparison of the DEGs by Gene Ontology (GO) analysis in different genotypes. It appears that BP, including brain development, RNA splicing, mRNA processing and gliogenesis (Additional file 7: Tables S16–18), are differentially affected by cell type-specific inactivation of MAGL, suggesting that regulation of biological processes by inhibition of 2-AG is cell type-specific.

### Transcriptomic changes in microglia and astrocytes are different in cell type-specific MAGL KO mice

**Fig. 2:**
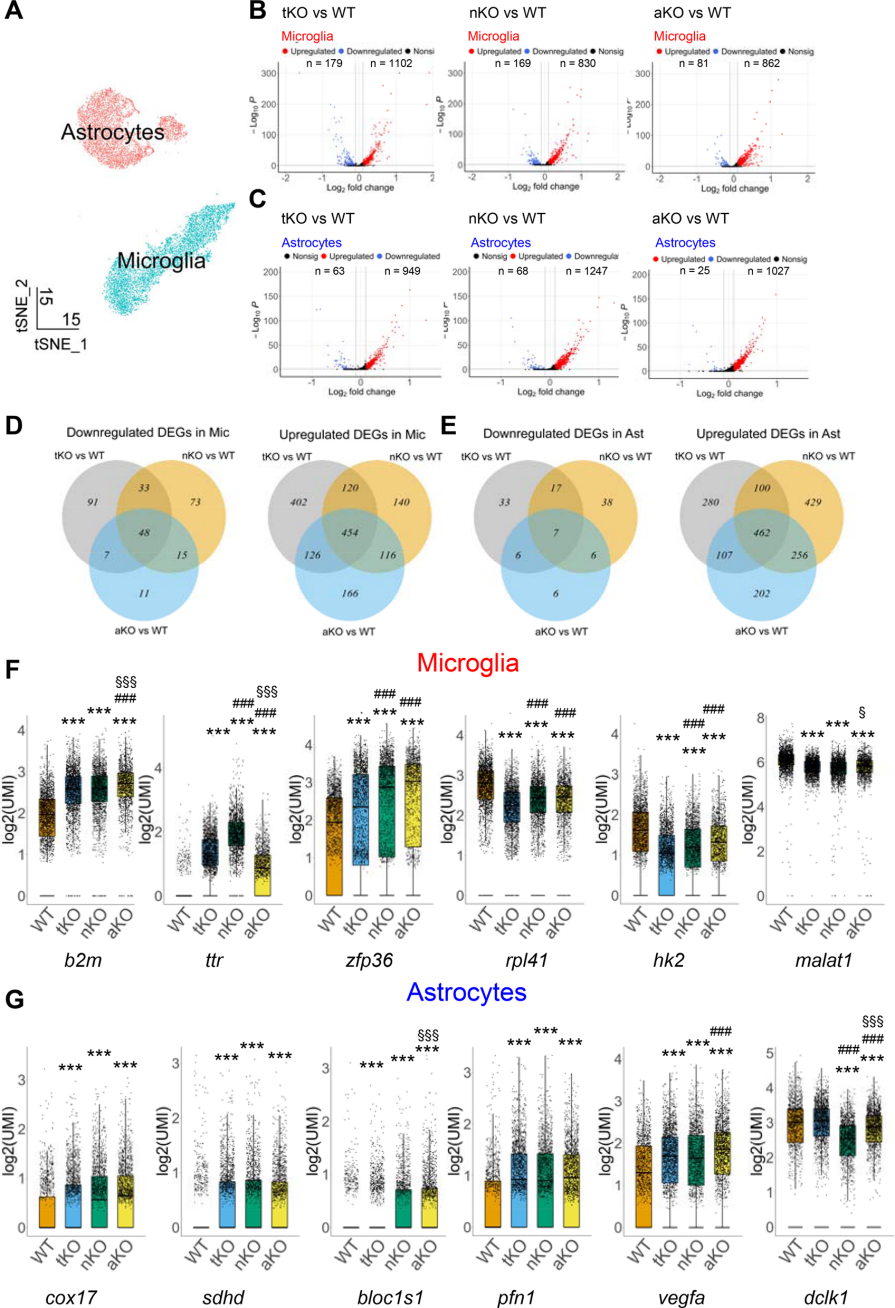
Cell type-specific inactivation of MAGL induces distinct gene expression profiles in microglia and astrocytes. **A** t-SNE plots of microglia and astrocytes clusters. Each colored dot represents a cell originated from WT, tKO, nKO or aKO mice. **B** Up- and down-regulated DEGs in microglia from different MAGL KO mice. **C** Up- and down-regulated DEGs in astrocytes from different MAGL KO mice. **D**, **E** The number of up- and down-regulated DEGs and their relationships in microglia and astrocytes from different genotypes. **F**, **G** Representative DEGs in microglia and astrocytes ***P < 0.0001 compared with WT; ^###^*P* < 0.001 compared with tKO; ^§^*P* < 0.05, ^§§§^*P* < 0.001 compared with nKO

Microglia and astrocytes in the brain are the main players in neuroinflammatory responses and in endocannabinoid signaling-mediated solution of neuroinflammation [37, 38]. To determine whether expression of genes in microglia and astrocytes is altered by inactivation of MAGL, we analyzed DEGs in microglia and astrocytes, which are identified based on known cell type-specific markers: *aif1*, *itgam* and *tmem119* for microglia; and *aqp4*, *gja1* and *slc1a2* for astrocytes (Additional file 2: Fig. S2 A–F). A total of 7536 microglia (2141 cells from WT, 2185 cells from tKO mice, 1857 cells from nKO mice, and 1353 cells from aKO mice) and 6769 astrocytes (1399, 1970, 1636, and 1764 astrocytes from WT, tKO, nKO, and aKO mice, respectively) passed the quality control and were selected for identification of DEGs (Fig. 2A). We identified that there are 1102 upregulated DEGs and 179 downregulated DEGs in microglia from tKO mice, 830 upregulated DEGs and 169 downregulated DEGs from nKO mice, 862 upregulated DEGs and 81 downregulated DEGs from aKO mice when compared to WT mice (Fig. 2B and Additional file 7: Tables S1–S3). In astrocytes, there are 949 upregulated DEGs and 63 downregulated DEGs in tKO mice, 1247 upregulated DEGs and 68 downregulated DEGs in nKO mice, and 1027 upregulated DEGs and 25 downregulated DEGs in aKO mice compared to WT mice (Fig. 2C and Additional file 7: Tables S4–S6).

To determine relationships between up- and down-regulated DEGs induced by cell type-specific inhibition of 2-AG metabolism, we generated Venn distribution plots. As shown in Fig. 2D, in microglia, there are 402 tKO-specific upregulated genes and 91 tKO-specific downregulated genes, 140 nKO-specific upregulated genes and 73 nKO-specific downregulated genes, and 166 aKO-specific upregulated genes and 11 aKO-specific downregulated genes (Fig. 2D). There are 454 upregulated and 48 downregulated genes overlapped among three groups. The numbers of overlapped DEGs between any two groups are as shown in Fig. 2D. In astrocytes, there are 280 tKO-specific upregulated genes and 33 tKO-specific downregulated genes, 429 nKO-specific upregulated genes and 38 downregulated genes, and 202 aKO-specific upregulated genes and 6 downregulated genes (Fig. 2E). There are 462 upregulated DEGs and 7 downregulated DEGs overlapped among three groups, which are likely expressed in astrocytes in MAGL KO mice (Fig. 2E). Figure 2F and G shows a few representative up- and down-regulated DEGs and their expression levels in microglia and astrocytes from different genotypes. Among top genes dysregulated in microglia, we observed a few important genes involved in learning and memory, including *ttr* and *b2m*, and genes that are crucial for cell apoptosis, including *zfp36*, *rpl41*, *hk2*, and *malat1* [44–49]. In astrocytes, a few dysregulated genes are associated with generation of precursor metabolites and energy (e.g., *cox17*, *sdhd*, and *bloc1s1*) and with cell migration (e.g., *pfn1*, *vegfa*, and *dclk1*) [50–55]. Interestingly, we noticed that up- and down-regulated DEGs in both microglia and astrocytes are more pronounced in aKO mice than in tKO or nKO mice, suggesting that inhibition of 2-AG degradation in astrocytes plays an unique role in regulation of cellular function.

To identify the biological processes (BP) potentially affected by the up- and down-regulated DEGs in different genotypes, we performed GO analysis. As shown in Additional file 3: Fig. S3A and B, altered DEGs in microglia from tKO mice are associated with the actin polymerization or depolymerization, leukocyte migration, cytoplasmic translation and ribosome biogenesis (Additional file 7: Table S13). In microglia of nKO mice, altered DEGs are likely involved in ATP metabolic process, response to endoplasmic reticulum stress, cytoplasmic translation and ribonucleoprotein and complex assembly (Additional file 7: Table S14). Altered DEGs in aKO mice participate in gliogenesis, response to oxidative stress, cytoplasmic translation, ribonucleoprotein complex biogenesis and response to interleukin-4 (Additional file 7: Table S15). Additional file 3: Fig. S3C and D displays altered DEGs in astrocytes. It appears that there are only upregulated DEGs in astrocytes from tKO mice, which may affect several biological processes, including glial cell differentiation, RNA splicing, cellular respiration and regulation of nervous system development (Additional file 7: Table S16). Altered DEGs from nKO mice may linked to regulation of neurogenesis, nervous system development, and neuron differentiation (Additional file 1: Table S17). DEGs in astrocytes from aKO mice may affect the cellular respiration, regulation of RNA splicing, synapse organization, central nervous system neuron development and axon genesis (Additional file 7: Table S18). These results suggest that cell type-specific inhibition of 2-AG metabolism may result in different transcriptome changes in microglia and astrocytes, which, in turn, regulate or control a variety of biological processes.

### Expression of cytokines or chemokines in microglia are promoted by inhibition of 2-AG metabolism in astrocytes

**Fig. 3:**
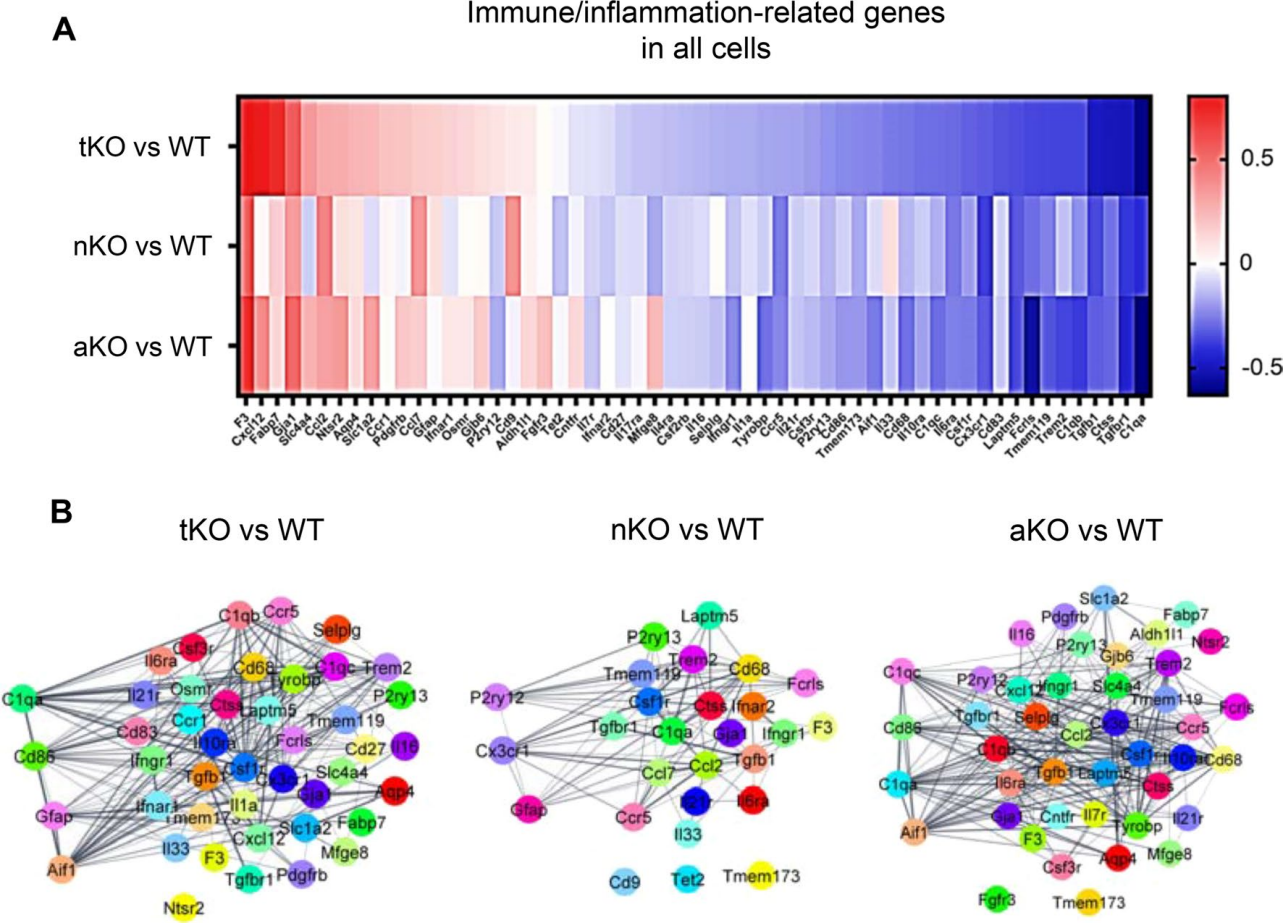
Differentially expressed immune- and inflammation-related genes (IGs) in all cells from MAGL tKO, nKO, and aKO mice. **A** Heatmap displays IGs in different genotypes. **B** Gene networks of IGs in different genotypes

Inactivation of MAGL produces significant anti-inflammatory and neuroprotective effects in both in vitro and in vivo [11–13, 21, 23–28, 56]. To determine whether inhibition of 2-AG metabolism alters expression of immune/inflammation-related genes, we analyzed immune/inflammation-related genes (IGs). As shown in Fig. 3A, many IGs are up- or down-regulated in MAGL KO mice, but the patterns of up- or down-regulated IGs are different between tKO, nKO, and aKO mice. For instance, there are 44 up- or down-regulated IGs in tKO mice, 27 in nKO mice, and 43 in aKO mice. These differentially expressed IGs display high confidence in the gene network supported by STRING analysis using Cytoscape software (Fig. 3B).

**Fig. 4:**
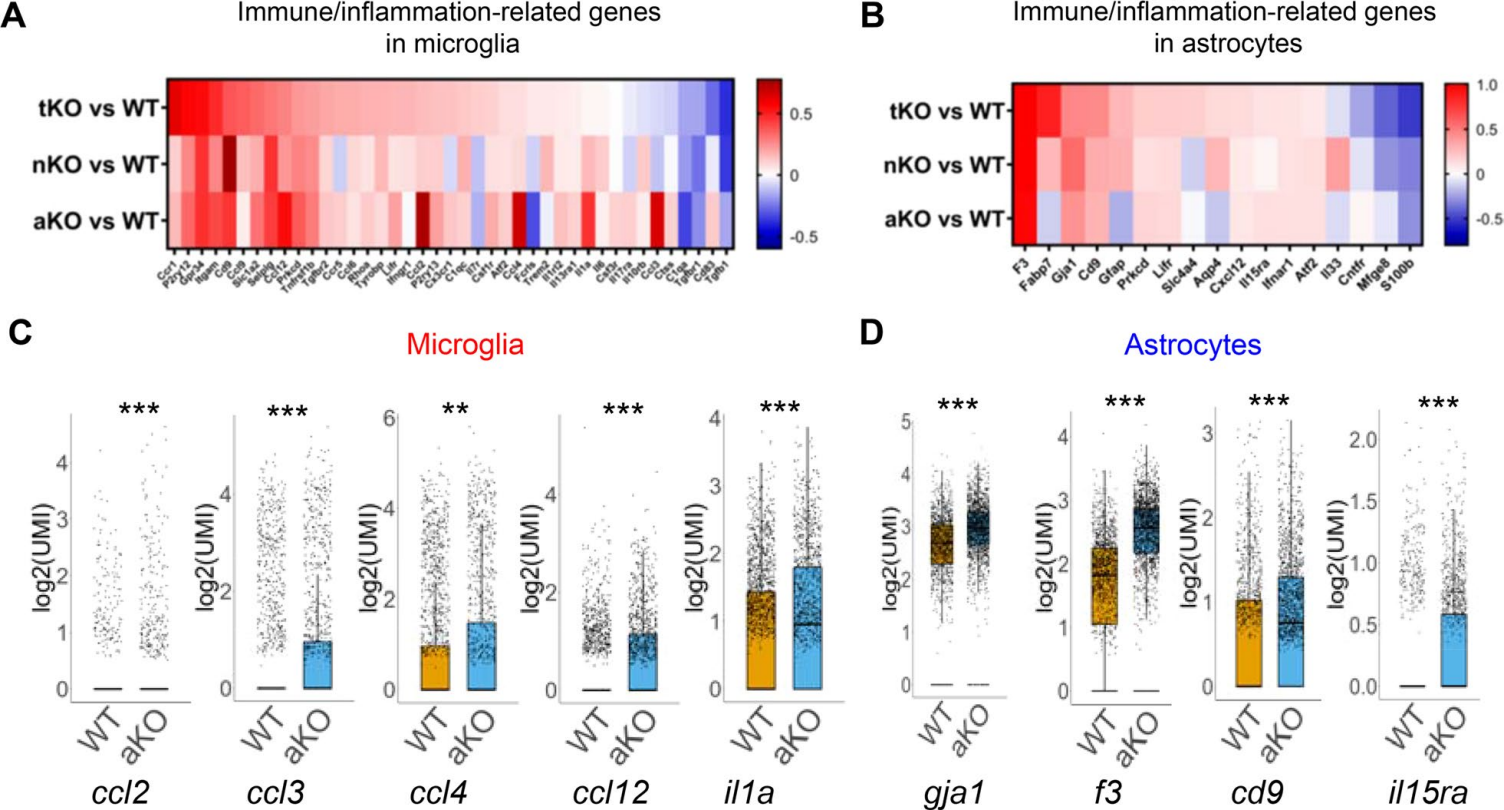
Differentially expressed immune- and inflammation-related genes (IGs) in microglia (**A**) and astrocytes (**B**) from MAGL tKO, nKO, and aKO mice. **C**, **D** Representative IGs differentially expressed in microglia and astrocytes from aKO mice. **P < 0.01, ***P < 0.001 compared with WT mice

**Fig. 5:**
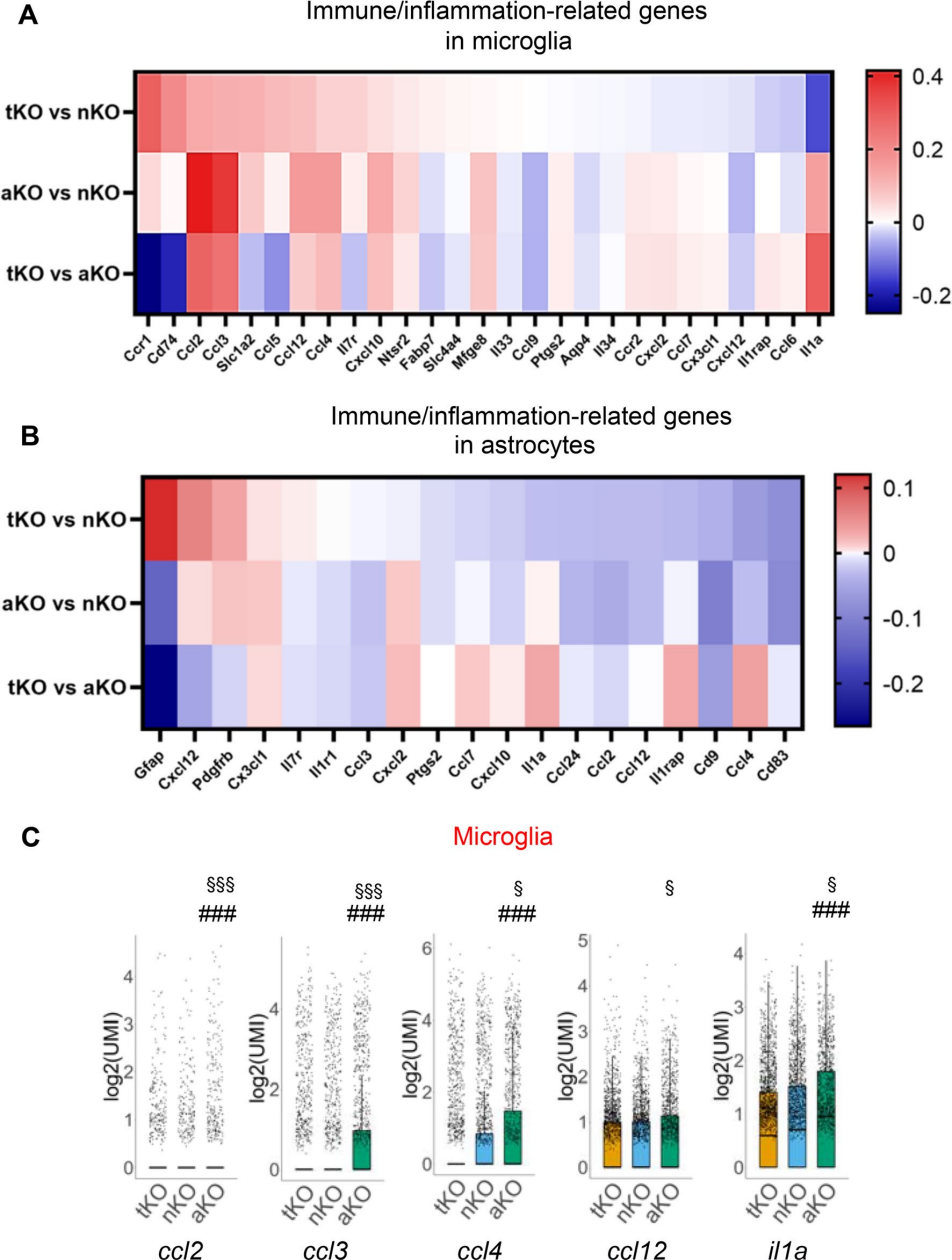
Comparison of differentially expressed immune/inflammation-related genes between tKO, nKO, and aKO mice. **A**, **B** Differentially expressed immune/inflammation-related genes in microglia and astrocytes between different genotypes. **C** Representative immune/inflammatory genes in microglia from different MAGL KO mice. ^###^*P* < 0.001 compared with tKO; ^§^*P* < 0.05, ^§§§^*P* < 0.001 compared with nKO

Next, we analyzed IGs in microglia and astrocytes from MAGL KO mice. As shown in Fig. 4A and B, the expression levels of many IGs are altered in microglia and astrocytes from MAGL KO mice compared to WT mice. In microglia, there are 27 altered IGs in tKO mice, 18 in nKO mice, and 30 in aKO mice. In astrocytes, there are 13 altered IGs in tKO, 12 in nKO mice, and 8 in aKO mice. Apparently, expression of IGs in glial cells is differentially up- or down-regulated, in particular, in microglia, in cell type-specific MAGL KO mice. Remarkably, expression levels of C-C motif chemokine ligand *ccl2*, *ccl3*, *ccl4*, *ccl12*, and *il1a*, which are important in innate immunity [57], are robustly upregulated in microglia from aKO mice (Fig. 4C), but expression of *ccl6* and *ccl9* is upregulated in tKO microglia (Fig. 4A). Interestingly, only very few cytokines are changed in astrocytes from MAGL KO mice (Fig. 4B). The significantly increased expression of genes that are associated with inflammation in astrocytes are *gja1*, *f3*, *cd9* and *il15ra* in aKO mice (Fig. 4D). We also observed that there are differences in changes in expression of IGs in microglia and astrocytes between different MAGL KO mice (Fig. 5A and B). In particular, expression of *ccl2*, *ccl3*, *ccl4*, *ccl12*, and *ii1a* in microglia are significantly upregulated in aKO mice when compared with tKO and nKO (Fig. 5C). These results indicate that cell type-specific inactivation of MAGL, especially inhibition of 2-AG metabolism in astrocytes, induces distinct gene expression profiles of chemokines in microglia, suggesting that inhibition of 2-AG metabolism in astrocytes promotes immune/inflammatory vigilance in microglia by signaling interacting between astrocytes and microglial cells.

We then used STRING analysis to identify predicted gene networks relevant to transcriptomes of microglia and astrocytes in different genotypes. In microglial gene networks, *cx3cr1ccl6*, *itgam*, *ccl9*, and *c1qc* are in specific confident centers in tKO mice (Additional file 4: Fig. S4A); *p2ry12*, *trem2, tyrobp*, and *il6* are in specific confident centers in nKO mice (Additional file 4: Fig. S4B); *ccl4, csf1r, cx3cr1*, *ccl2* and *itgam* are in specific confident centers in aKO mice (Additional file 4: Fig. S4C). Our results suggest that *itgam* and *cxcl12* mice are the important genes in the confident centers primarily in microglial gene networks when MAGL is inactivated.

### MAGL inactivation regulates expression of genes involved in oxidative stress and neural functions

**Fig. 6:**
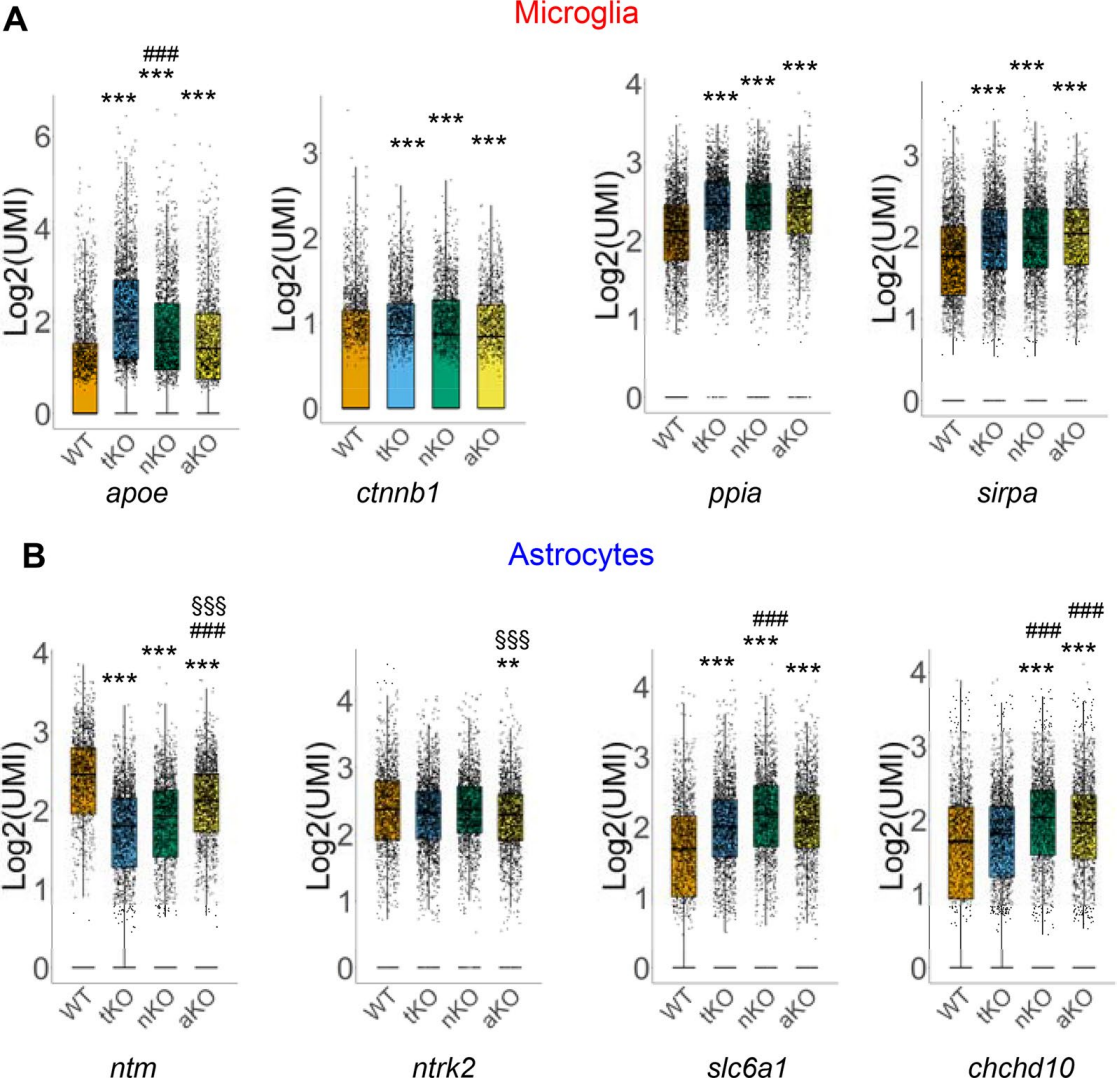
**A** Differentially expressed genes involved in “Oxidative response” in microglia from different genotypes. **B** Differentially expressed genes involved in “Neural function” in astrocytes. **P < 0.01; ***P < 0.001 compared with WT; ^###^*P* < 0.001 compared with tKO; ^§§§^*P* < 0.001 compared with nKO

Previous studies reveal that 2-AG protects neurons against oxidative stress and regulates functions of the nervous system [58]. In analyzing immune- and inflammation-related DEGs in MAGL KO mice, we also noticed significant changes in expression of genes regulating oxidative stress and neuronal functions. As shown in Fig. 6 and Additional file 5: Fig. S5, genes (e.g., *fos*, *cst3*, *txnip*, *prdx1*, *ndufs8*, *cfl1*, *atp2a2*, *apoe*, *ppia*, *rhob*, *selenok*, and *sirpa*) that are important for responses to oxidative stress are significantly changed in microglia in MAGL KO mice. In addition, we observed changes of genes (e.g., *ntrk2*, *gpm6a*, *ntm*, *slc6a1*, *epha5*, *pax6*, *cadm1*, *ndrg2*, *dclk1*, *chchd10*, *syne1*, and *tnik)* that regulate neural functions in astrocytes. Changes in expression levels of these DEGs in microglia are similar in all genotypes. For example, expression of *apoe*, *ctnnb1*, *sirpa*, and *ppia* in microglia is upregulated in tKO, nKO, and aKO mice. However, changes in expression levels of the DEGs in astrocytes are heterogeneous. For instance, expression of *epha5, pax6, syne1*, and *tnik* in astrocytes is altered only in nKO mice. In contrast, changes in expression of *ntrk2* and *cadm1* in astrocytes are only seen aKO mice. These results suggest that selective inactivation of MAGL, which changes expression of genes in microglia and astrocytes, may differentially regulate oxidative stress and neuronal functions.

### Regulation of intercellular communications by inactivation of MAGL

**Fig. 7:**
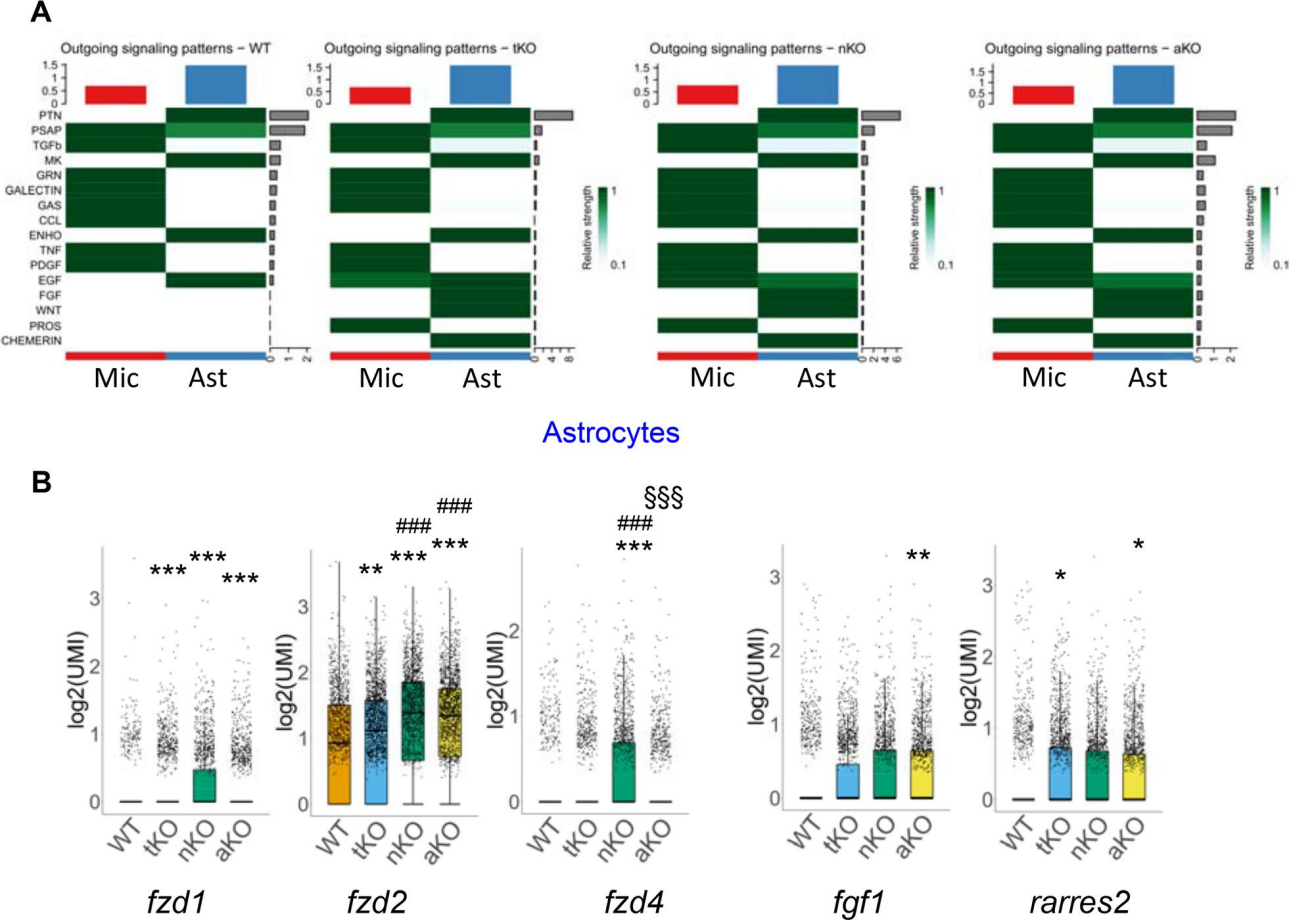
Enhanced cell–cell communications in cell type-specific MAGL KO mice. **A** Signaling interactions are strengthened in microglia and astrocytes by inactivation of MAGL. **B** Representative ligands or receptors that are altered in astrocytes from different genotypes. *P < 0.05, **P < 0.01, ***P < 0.001 compared with WT; ^###^*P* < 0.001 compared with tKO; ^§§§^*P* < 0.001 compared with nKO

Expression of genes and functions in individual cells are not only affected by intracellular molecules, but also regulated or modulated by intercellular communications [59]. Based on the data shown above, inhibition of 2-AG metabolism in astrocytes significantly alters expression of genes in microglia, indicating signaling interactions between microglia and astrocytes in aKO mice. To this end, we used Cellchat package in R (4.0.4) to analyze signaling molecules that are changed by cell type-specific inactivation of MAGL in microglia and astrocytes. Since intercellular communications are primarily regulated by ligand–receptor pairs, we explored the number of interactions (ligand–receptor pair) and interaction strengths in different genotypes. We found that there are many significant changes in ligand–receptor pairs MAGL KO mice. These ligand–receptor pairs are categorized into several different signaling pathways, including PTN, PSAP, TGFb, MK, GRN, CALCTIN, GAS, CCL, ENHO, TNF, PDGF, EGF, FGF, WNT, PROS, and CHEMERIN (Fig. 7A). It appears that the interaction strength of the CCL pathway is attenuated in microglia from tKO mice, but those of EGF and PROS pathways are boosted in microglia of tKO, nKO and aKO mice (Fig. 7A). In astrocytes, relative strengths of FGF, WNT and CHEMERIN pathways are enhanced in conditional KO mice (Fig. 7A).

We further analyzed expression levels of ligands or receptors in these signaling pathways. As shown in Fig. 7B, expression of some of ligands and receptors is upregulated in astrocytes. For example, expression levels of *fzd1*, *fzd2*, and *fzd4,* which are involved in Wnt signaling pathways, in astrocytes are significantly increased in tKO, nKO and aKO mice. In addition, expression levels of *fgf1* and *rarres2* in astrocytes are elevated in aKO mice. Similarly, expressions of *pros1* and *tgfa* are elevated in microglia from all the genotypes, while expression of *ncl* is increased only in microglia of nKO mice (Additional file 6: Fig. S6A).

**Fig. 8:**
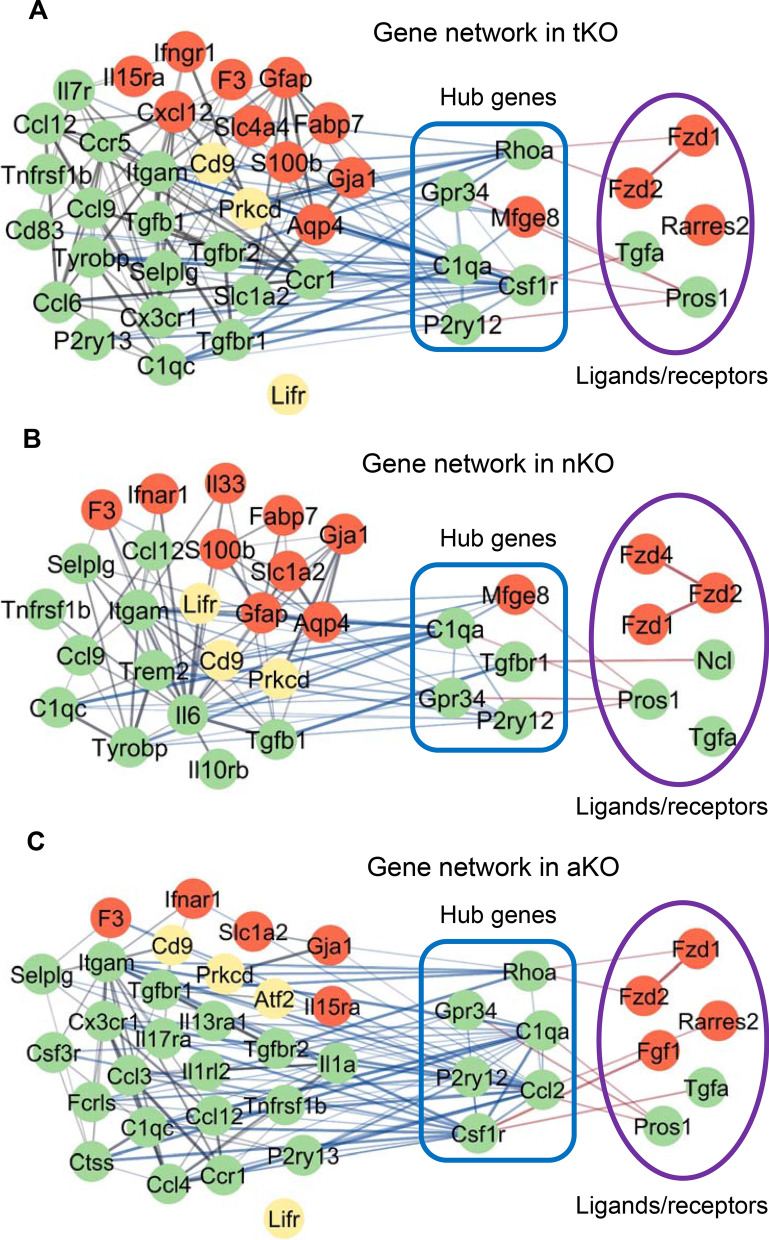
Gene networks between astrocytes and microglia from different genotypes. **A** Interactions of DEGs and IGs in astrocytes and microglia in tKO mice. **B** Interactions of DEGs and IGs in microglia and astrocytes in nKO mice. **C** Interactions of DEGs and IGs in astrocytes and microglia in aKO mice. Node color: green indicates DEGs in microglia; red indicates DEGs in astrocytes; yellow indicates DEGs in both microglia and astrocytes

We hypothesized that these ligands or receptors likely play an important role in regulating expression of immune/inflammation-related genes. To this end, we used STRING analysis to reveal interactions between IGs and ligands/receptors. As shown in Fig. 8, these ligands or receptors may interact with multiple genes, including *p2ry12*, *c1qa*, *csf1r*, *gpr34*, *mfge8*, *tgfb1*, *and ccl2*. These ligands or receptors may also regulate other IGs indirectly via these hub genes (Fig. 8). Interestingly, *fzd1* and *fzd2* in astrocytes could modulate *itgam*, *prkcd*, and *tgfbr1* in microglia via the hub gene *rhoa* in aKO and tKO mice (Fig. 8A and C). In aKO mice, *fgf1* is crucial ligand in astrocytes, which may regulate IGs in microglia (Fig. 8C). Additional file 6: Fig. S6B and 6C display gene networks, which indicate ligands and receptors interacting with DEGs in microglial and astrocytes, which modulate “Response to oxidative stress” and neuronal functions in aKO mice. Particularly, receptors in Wnt signaling pathways interact with several hub genes in all gene networks.

Our results provide evidence that inactivation of MAGL induces changes in expression of ligands and their receptors, which may further regulate expression of immune- and inflammation-related genes in microglia and astrocytes. MAGL inactivation-induced changes in expression of genes involved in Wnt signaling pathways in astrocytes likely contributes to these intercellular communications.

## Discussion

Previous studies demonstrated that 2-AG or inactivation of MAGL produces anti-inflammatory and neuroprotective effects in vitro and in animal models of neurodegenerative diseases [11–13, 21, 23–28, 39, 60]. However, little is known about the molecular mechanisms underlying the beneficial effects produced by inactivation of MAGL. In the present study, we used single-cell RNA sequencing analysis to profile expression of genes in microglia and astrocytes from cell type-specific MAGL knockout mice, as glial cells are central players in maintaining brain homeostasis and in brain disorders. We observed that cell type-specific MAGL KO mice display discrete gene expression profiles. Importantly, inactivation of MAGL results in robust changes in expression of immune- and inflammation-related genes in microglia and astrocytes. Remarkably, upregulated expression of immune genes in microglia is more pronounced in mice lacking MAG in astrocytes, indicating that inhibition of 2-AG metabolism in astrocytes promotes glial immunity and vigilance. In addition, expression of genes that regulate other cellular functions and Wnt signaling in astrocytes is altered in MAGL KO mice. These changes are likely associated with increased expression levels of ligands and receptors in microglia and astrocytes of MAGL KO mice. Our results provide transcriptomic evidence that inactivation of MAGL alters expression of genes that regulate immune, inflammation, oxidative stress, and neuronal functions in microglia and astrocytes. Upregulation of inflammation-related genes by inhibition of 2-AG metabolism in astrocytes suggests that anti-inflammatory and neuroprotective effects produced by inactivation of MAGL in neurodegenerative diseases are likely through promoting tonic levels of vigilance or alertness in microglia and astrocytes, particularly in microglia, which enable glial cells to react rapidly to harmful insults or attacks.

MAGL is a key enzyme degrading 2-AG in the brain [19, 20, 22, 23]. While 2-AG is capable of resolving neuroinflammation and protecting neurons from harmful insults [11–13, 15], its metabolites eicosanoids are proinflammatory mediators [61, 62], suggesting that inactivation of MAGL produces dual beneficial effects, boosting anti-inflammatory 2-AG and concurrently declining inflammatory eicosanoids [2, 20, 22, 23, 31, 63]. Inflammation is a common mechanism of disease [34]. Therefore, resolution of neuroinflammation by inactivation of MAGL plays an important role in neurodegenerative diseases. We found in the present study that immune- and inflammation-related genes are robustly upregulated in microglial and astrocytes from MAGL KO mice, suggesting that inactivation of MAGL promotes tonic levels of vigilance in microglia and astrocytes, which are important for glial cells to maintain homeostasis in the brain by rapidly reacting to harmful insults. In particular, expression of c-c motif chemokines in microglia, including *ccl2*, *ccl3*, *ccl4*, *ccl12* and *il1a*, which are important in innate immunity [57], is more pronounced in aKO mice when compared with tKO or nKO mice. These data suggesting that promoting glial alertness or strengthening glia ability to prevent danger in the brain by inactivation of MAGL is cell type-specific and that inhibition of 2-AG metabolism in astrocytes is crucial in enhancing microglial immunity and phagocytosis as well as resolving or terminating neuroinflammation in neurodegenerative diseases.

Interestingly, we identified that there are more upregulated DEGs in tKO and aKO mice, while there are more downregulated DEGs in nKO mice. This is likely associated with the different roles of 2-AG signaling and its metabolites played in neurons and astrocytes. For instance, 2-AG is a retrograde messenger in regulation of synaptic transmission and plasticity at both GABAergic and glutamatergic synapses [3–9]. It is likely that 2-AG generated in neurons is responsible for modulation of synaptic activity, while 2-AG synthesized in astrocytes is responsible for termination or resolution of inflammation. This is supported by the findings from a previous study where the authors showed that arachidonic acid and its metabolites prostaglandins, which are proinflammatory, are primarily derived from 2-AG in astrocytes and that pharmacological or genetic inactivation of MAGL in astrocytes greatly reduces amount of prostaglandins [22]. This means that 2-AG generated from astrocytes participates in inflammatory responses. Therefore, these factors may be a possible reason why there are more upregulate DEG in tKO and aKO mice, while there are more downregulated DEG in nKO mice. Our recent study revealed that inactivation of MAGL in astrocytes, but not in neurons, reduces TBI-induced neuroinflammation, including expression of cytokines and reactivity of astrocytes and microglia [36, 39], which may underscore the consequences of these discrepancies among aKO, nKO, and tKO mice.

We also observed that several genes that are involved in oxidative stress are upregulated in microglia from MAGL KO mice. For instance, expression of *apoe*, *cst3* and *fos* in microglia is upregulated. These genes have been shown to play important roles in defense of oxidative stress-induced cell death [64–66]. Upregulation of these genes may underlie decrements in oxidative stress-induced death by MAGL inactivation in AD model animals [25]. In addition, astrocytic expression of *slc6a1*, which regulate reuptake of GABA in neurons and glia [67], and *chchd10*, which have protective role in mitochondrial and synaptic integrity [68], is upregulated in MAGL KO mice, suggesting that inhibition of 2-AG degradation may also participate in regulation of neuronal functions.

Since MAGL in microglia does not play a significant role in hydrolyzing 2-AG [22, 40], we did not generate KO mice deficient in MAGL in microglia. However, our data reveal that there are robust changes in expression of many genes, especially immune- and inflammation-related genes in microglia. This suggests that there exist intercellular signaling communications between neurons, astrocytes and microglia, resulting in changes in gene expression profiles in microglia in MAGL KO mice. In particular, expression of immune genes in microglia is upregulated in aKO mice, indicating that limiting 2-AG degradation in astrocytes promotes the immune alertness in microglia through signaling interactions between astrocytes and microglia. Indeed, we found that increased expression of *fzd1* and *fzd2* in astrocytes that are involved in Wnt signaling pathways, which regulates *itgam*, *prkcd*, and *tgfbr1* in microglia via the hub gene *rhoa* in tKO and aKO mice. In addition, expression of *ccl3*, *ccl4*, *fgf1*, *tgfα*, and *ccl12* in microglia or astrocytes is significantly altered in tKO, nKO, and aKO, especially, in aKO mice. These genes and those involved in Wnt signaling in microglia and astrocytes participate in immune/inflammatory signaling pathways. For instance, *ccl2* and *il1α* are highly expressed in microglia and are key mediators for microglia activation and proliferation [69–72]. However, *ccl2* also regulates neuronal excitability and synaptic transmission [73], whereas *il1a* has been shown to be a “dual-function” molecule that is neuroprotective and promotes protective microglial immunity [74, 75]. Besides, microglia-derived *il1a* can also impact on astrocytic function [76]. A recent study showed that *tgfα* secreted from microglia binds to *erbb1* receptors in astrocytes and inhibits experimental autoimmune encephalomyelitis development [77]. Therefore, discrete gene expression profiles displayed in cell type-specific MAGL KO mice are likely associated with intercellular signaling interactions or communications between neurons, astrocytes, and microglia. While we did not measure the level and expression of MAGL in microglial cells from aKO and nKO mice in the present study, we cannot exclude the possibility that some of the changes in expression of genes may result from genetic compensation by microglial-MAGL in aKO or nKO mice.

While the 10 × genomics technology has been widely used in transcriptomic analysis of gene expression at single cell levels, it does have some limitation. For instance, compared with other RNA sequencing technologies (e.g., Smart-seq2), 10 × -based data display relatively high noise for mRNA signals and a dropout problem for genes with lower expression levels. In addition, 10 × platform might detect less genes in individual cells [78]. However, the 10 × platform still displays a huge advantage for its high-throughout single-cell analysis and is an innovative solution for robust, scalable single-cell analysis and opens the door for analysis of single-cell genomics, transcriptomics and epigenomics. No doubt, no other techniques currently can replace the 10 × genomics technology for single-cell RNA sequencing. Another potential issue we need to mention here is the method to dissociate cells from adult animals. Enzymatic dissociation of brain cells at 37 °C is widely used in adult animals for single-cell RNA sequencing studies. However, a recent study showed that enzymatic digestion at 37 °C might result in the activation of inflammatory and immune genes when compared with that using mechanical dissociation at 4 °C [79]. While enzymatic digestion for dissociation of cells may yield biological artifacts as shown in this study [79], lower temperature and mechanical stretch may also change expression of genes. In particular, mechanosensitive ion channels in glial cells are able to sense vibration, pressure, or mechanical stretch, which may activate or inactivate expression of certain genes. Therefore, caution should be exercised in interpreting single-cell RNA sequencing data in brain cells using different cell isolation methods.

## Conclusions

Our single-cell transcriptomic analysis reveals distinct gene expression profiles in the brain of cell type-specific MAGL KO mice. Importantly, immune- and inflammation-related genes are significantly upregulated in microglia and astrocytes in tKO, nKO, and aKO, but the upregulation in microglia is predominant in aKO mice, suggesting that inhibition 2-AG metabolism in astrocytes promotes the basal tone of immune vigilance and activates the glial immunity system, which may contribute to anti-inflammatory and neuroprotective effects observed by inactivation of MAGL in neurodegenerative diseases.

## Supplementary Information

**Additional file 1.** Venn plots for upregulated differentially expressed genes (DEGs).

**Additional file 2.** Clusters of specific expression of known cell markers.

**Additional file 3.** Gene Ontology (GO) analysis of biological process (BP) terms of DEGs in microglia and astrocytes from different genotypes.

**Additional file 4.** Gene networks for immune/inflammation-related genes (IGs) in microglia.

**Additional file 5.** Representative differentially expressed genes (DEGs) involved in “Oxidative response” signaling pathways in microglia and representative DEGs in “Neuronal functions” signaling pathways in astrocytes.

**Additional file 6.** Ligands/receptors and their interactions with DEGs involved in “response to oxidative stress” signaling pathway and neural functions.

**Additional file 7.** ScRNA-supplementary tables.

## Data Availability

The data supporting the findings of this manuscript are presented within the manuscript. The singe-cell RNA-seq data have been deposited in the NCBI Gene Expression Omnibus under accession number “GSE178226”.

## Abbreviations

2-AG: 2-Arachidonoylglycerol
MAGL: Monoacylglycerol lipase
tKO: Total MAGL knockout
nKO: Neuronal MAGL knockout
aKO: Astrocytic MAGL knockout
AA: Arachidonic acid
PGs: Prostaglandins
COX-2: Cyclooxygenase-2
ABHD6/12: α/β Hydrolase domain-containing protein 6 and 12
ScRNA-seq: Single-cell RNA sequencing
DEG: Differential expressed gene
t-SNE: T-Distributed stochastic neighbor embedding
GO: Gene ontology
IGs: Immune/inflammation-related genes
